# Transplantation sites for porcine islets

**DOI:** 10.1007/s00125-017-4363-7

**Published:** 2017-07-21

**Authors:** Rebecca A. Stokes, Denbigh M. Simond, Heather Burns, Anita T. Patel, Philip J. O’Connell, Jenny E. Gunton, Wayne J. Hawthorne

**Affiliations:** 1 Centre for Diabetes, Obesity & Endocrinology, The Westmead Institute for Medical Research (WIMR), The University of Sydney, Sydney, NSW Australia; 2 Diabetes and Transcription Factors Group, Garvan Institute of Medical Research (GIMR), Sydney, NSW Australia; 3 National Pancreas and Islet Transplant Unit, University of Sydney, Westmead Hospital, Sydney, NSW Australia; 4 Faculty of Medicine, University of Sydney, Sydney, NSW Australia; 5 Centre for Transplant and Renal Research, The Westmead Institute for Medical Research (WIMR), The University of Sydney, Sydney, NSW 2145 Australia; 6 St Vincent’s Clinical School, University of New South Wales, Sydney, NSW Australia

**Keywords:** Experimental diabetes mellitus, Islet transplantation, Xenotransplantation

## Abstract

**Aims/hypothesis:**

Xenotransplantation has great potential to provide beta cell replacement and thereby provide a cure for large numbers of people with type 1 diabetes. Crucial to the success of xenotransplantation is establishment of the most viable sites for transplantation.

**Methods:**

We compared porcine islet tissue transplanted into kidney, liver and spleen in pig recipients as assessed by blood glucose levels and IVGTT.

**Results:**

Kidney was the superior site for porcine islet tissue transplantation, followed by liver then spleen. This was demonstrated by IVGTTs showing significant difference between the peak glucose levels: 22.8 ± 2.9 mmol/l for kidney compared with 26.8 ± 1.3 mmol/l for spleen and 24.7 ± 1.7 mmol/l for liver.

**Conclusions/interpretation:**

Kidney grafts are not as feasible in humans and liver results were relatively poorer than spleen. For islet transplantation to be viable and successful in the longer term, there remains a need for future investigation of alternative sites.

## Introduction

The shortage of human donor pancreases has an impact on people with type 1 diabetes who require an organ transplant. Major technological advances make xenotransplantation a potential source from which to supply enough beta cell tissue to cure a large proportion of people with type 1 diabetes [1]. However, the optimal graft site has not been clearly elucidated. In humans, the current clinical site is infusion into the portal vein [2]. However, there are potential disadvantages such as portal hypertension, bleeding, thrombosis, ischaemia, inability to perform protocol biopsies and restrictions and, to date, long-term success rates lag behind those of whole-pancreas transplantation [3].

Several sites have been investigated including muscle [4], spleen [5], portal vein [6] and the kidney subcapsular space [7]. In our companion paper (Stokes et al [8]), we compare outcomes for mouse and human islets transplanted into a range of sites.

Porcine tissue is the most promising tissue source for future human xenotransplantation. Our study compares outcomes for porcine islet tissue placed in the kidney, liver and spleen.

## Methods

### Ethics

Animal studies were approved by the Western Sydney Local Health District Animal Ethics Committee. NIH guidelines regarding reporting of experimental conditions were followed.

### Animals

Recipients were 6-month-old male Westran pigs (Westmead Hospital, Westmead, NSW, Australia). Westran pigs are a highly inbred strain (>13 generations of sibling mating). Donors were Westran fetal piglets at 70–90 days gestation. Recipient pigs were housed at the Westmead Hospital Research Centre using approved husbandry and ethical standards [9].

### Induction of diabetes in pigs

Recipient pigs underwent a total native pancreatectomy performed via midline incision under general anaesthesia between day 96 and 100 after transplantation. The duodenum was preserved. All pancreatectomised pigs received pancreatic enzyme replacement (Cotazym-S Forte capsules; Organon Canada, Scarborough, ON, Canada) daily.

### Porcine islet isolation and transplantation

Porcine pancreatic islets were isolated from 70–90-day-old fetal pig pancreases as described previously [9]. Islet tissue was transplanted from 72 piglets into 18 recipient adults (four donors per recipient). The transplant sites were beneath the capsule of the liver, kidney or spleen. Equivalent amounts of islet tissue were transplanted into each of the organs and there were no macroscopic differences at transplantation or at the time of biopsy. For transplantation, a mini-laparotomy was performed on recipient Westran pigs under general anaesthetic to expose the organs. An incision was made in the capsule of the transplanted organ to permit insertion of a 14-gauge cannula and the islets were injected as the cannula was slowly withdrawn. A 5.0 Prolene purse-string suture was used to close the injection site. Recipient pigs were transplanted with islets at a dose of ≥5000 islet equivalents (IEQ) per kg body weight, which is analogous to the usual human situation. The native pancreas was removed at operation between days 96 and 100 as above. Grafts were biopsied 120 days after transplantation (i.e. 20 days after native pancreatectomy). No immunosuppression was provided. Islet samples were randomised for both site and recipient. The experimenter was both islet isolator and surgeon and therefore unable to be blind to group assignment; hence randomisation was carried out where possible. The same experimenter was blind to outcome assessment due to randomisation.

### IVGTT

In the pigs, IVGTTs were performed as previously described [9]. Central venous catheters (CVCS; Arrow International, Reading, PA, USA) were used to cannulate the jugular vein and glucose was injected into a cephalic vein at a dose of 1 g/kg body weight. Blood samples were obtained before injection and at the times shown. Blood glucose levels (BGLs) were measured using a Vitros 5.1/FS system (Ortho-Clinical Diagnostics, Rochester, NY, USA) and porcine insulin concentrations using a porcine-specific RIA (Linco Research, St Louis, MO, USA).

### Immunohistochemistry

Fetal pig islet graft biopsies were fixed in 10% neutral buffered formalin and 5 μm sections were cut from each paraffin block. Slides were stained using specific monoclonal antibodies for insulin, chromogranin, somatostatin and glucagon (Zymed Laboratories, South San Francisco, CA, USA). Secondary antibodies used were Idetect Super Stain System HRP (ID Labs, London, ON, Canada), and counterstaining was with haematoxylin. For CD-31, the primary antibody was mouse anti-CD31 monoclonal diluted 1:100 (Dako, Carpinteria, CA, USA) and the secondary antibody was anti-mouse MACH 2 HRP-polymer (Biocare, Pacheco, CA, USA). Detection was carried out using a Biocare peroxidase substrate containing 3, 3-diaminobenzidine (brown) and then counterstaining with haematoxylin. Images were taken with a Leica DM 5500 microscope and Zeiss AxioVision software (Oberkochen, Germany).

### Statistical analysis

The *k* value for the porcine IVGTT describes the time required for glucose to clear the circulation and was calculated using the formula *k* = 0.693/*t*_½_ × 100, where *t*_½_ is the time required for plasma glucose to reach one half of the zero-time concentration. No animals or results were excluded.

## Results

### In pigs, transplanted porcine islets were functional and maintained normal BGLs regardless of site

**Fig. 1:**
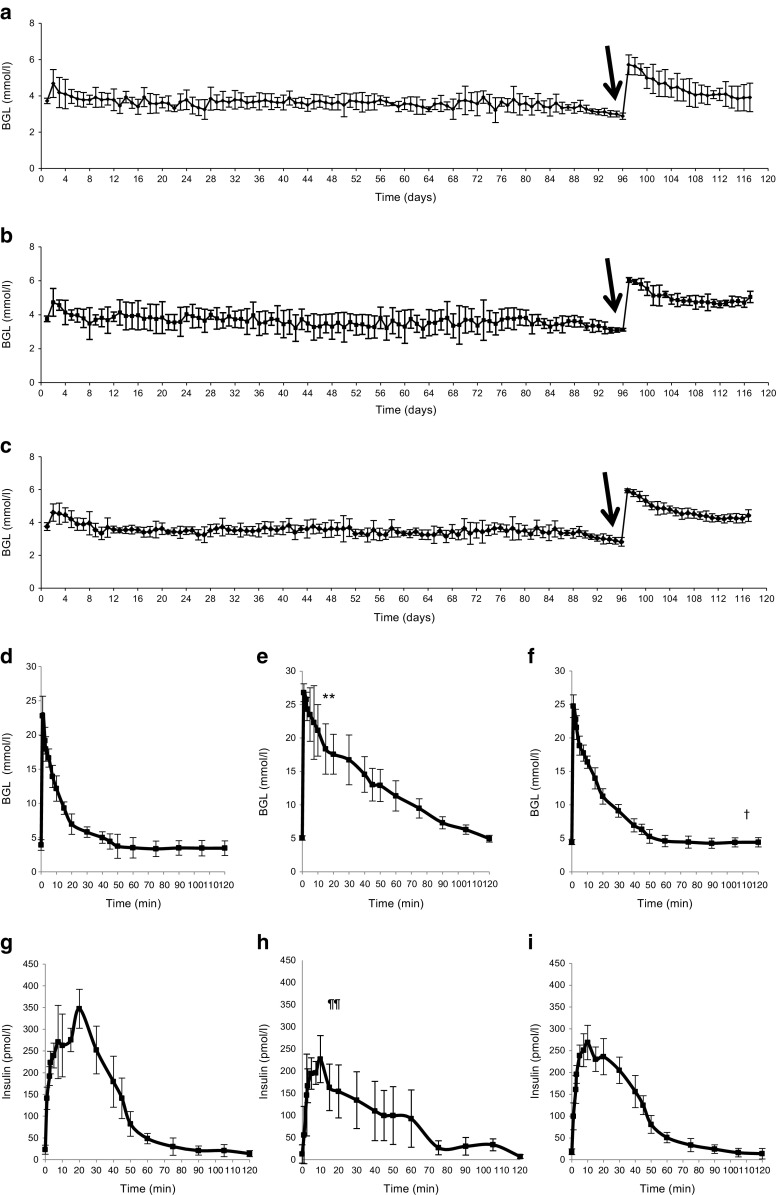
Porcine islet transplants and graft function. (**a**–**c**) Daily blood glucose levels (BGLs) at 120 days post-transplant were superior in pigs with kidney-transplanted grafts (*n* = 6) (**a**) compared with those with liver capsule-transplanted grafts (*n* = 6) (**c**) and the most inferior spleen-transplanted grafts (*n* = 6) (*p* = 0.007) (**b**). Arrow indicates native pancreatectomy. (**d–f**) Islet function was determined by IVGTT. Blood glucose response was superior in pigs with kidney-transplanted grafts (*n* = 6) (**d**) compared with spleen-transplanted grafts (*n* = 6) (**e**); peak BGL, **p < 0.01 for spleen vs kidney graft recipients, and no different in pigs with liver capsule-transplanted grafts (*n* = 6) (**f**). †*p* < 0.05 for time to return to baseline glucose vs both kidney and liver-graft recipients. Recipients with kidney-transplanted grafts had the fastest return to basal glucose of 60 min, followed by liver capsule-transplanted recipients at 72 min and spleen-transplanted recipients at 118 min. (**g**–**i**) The insulin response (measured by RIA) was better in pigs with kidney-transplanted grafts (**g**) than in those with spleen-transplanted grafts (**h**) (^¶¶^ *p* < 0.01); there was a lower response in recipients of islet transplants into liver capsule (**i**) (not statistically significant, *p* = 0.068 vs kidney). The kidney-transplanted graft had slower initial insulin peak and significant second-phase insulin release

Subcapsular islet allotransplants showed that all pigs had functional grafts that normalised BGLs, regardless of the transplant site, at 120 days after transplantation. Mean fasting BGL of recipients at 120 days after transplantation of islets into the various sites was 3.9 ± 0.8 mmol/l for kidney, 5.0 ± 0.3 mmol/l for spleen and 4.4 ± 0.4 mmol/l for liver (Fig. 1a–c). Thus, all groups had normal fasting BGLs. However, the fasting BGL of recipients transplanted at the spleen site was significantly higher than in that of those transplanted into the kidney (*p* = 0.007).

BGLs during the IVGTTs showed significant differences between the peak glucose levels (Fig. 1d–f). Results for the kidney-transplanted recipients were superior to those for the spleen-transplanted recipients: peak glucose was 22.8 ± 2.9 mmol/l for kidney vs 26.8 ± 1.3 mmol/l for spleen (*p* = 0.008). Peak glucose was 24.7 ± 1.7 mmol/l in liver-transplanted recipients, not significantly different from that in kidney-transplanted recipients. The time to return to baseline BGL was faster in recipients of islets transplanted into kidney (60 min) and liver (72 min) than in spleen-site recipients (118 min, both *p* < 0.05). The *k* value was 0.77 ± 0.08%/min in the kidney-site transplant recipients vs 0.48 ± 0.04%/min in spleen-site transplant recipients and 0.66 ± 0.07%/min in liver-site transplant recipients.

During the IVGTTs, the insulin response was higher in the recipients of transplants into the kidney vs spleen (Fig. 1g–i, *p* < 0.01 ANOVArm). The insulin response was lower in liver-site transplant recipients than in kidney-site transplant recipients but the difference did not reach statistical significance (*p* = 0.068 ANOVArm). The time to initial peak insulin was longer in recipients transplanted at the kidney site, with a significant second-phase insulin release (increased insulin release after 10 min) only occurring in the kidney-site transplant recipients when compared with both liver and spleen (both *p* < 0.001).

**Fig. 2:**
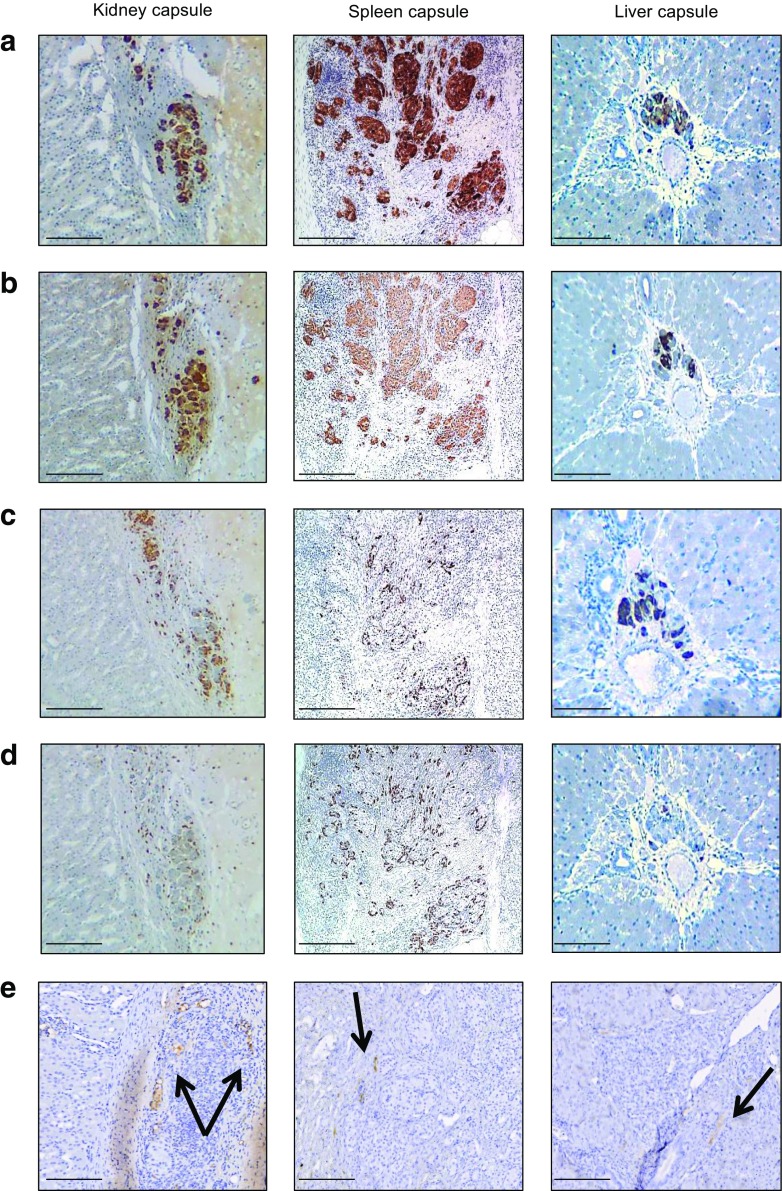
Histological analysis of porcine islet transplants. Graft biopsies were sectioned and stained as described in Methods. (**a**) Porcine islet allografts under the kidney, spleen or liver capsule 120 days after transplantation showed strong staining for chromogranin A. (**b**–**d**) Approximately 80% of islet cells showed strong insulin (**b**), glucagon (**c**) and somatostatin staining (**d**). (**e**) The intensity of CD31 endothelial staining was similar in all tissue types. Scale bars, 400 μm (for figures under the Kidney and Spleen columns) and 200 μm (for figures under the Liver capsule column). Black arrows indicate the site of CD31-positive endothelial staining

All recipient sites had macroscopically visible areas of transplanted pancreatic tissue under the surface of the organ (Fig. 2). Biopsies showed that porcine islet grafts survived without signs of rejection in all sites, with beta cells present and staining strongly for insulin, glucagon and somatostatin, regardless of the site. At 120 days, there was distinct endocrine tissue present with strong chromogranin A staining at all sites (Fig. 2a). Approximately 80% of cells stained strongly for insulin (Fig. 2b), glucagon (Fig. 2c) and somatostatin (Fig. 2d). Endothelial CD31 cell staining showed similar revascularisation in all tissues (Fig. 2e).

Overall, our results show that the best outcome was achieved using the kidney capsule as a site for fetal pig pancreatic islet transplantation, followed by the liver.

## Discussion

Xenotransplantation using porcine pancreatic islets has the potential to alleviate donor shortages and to provide a nearly unlimited source of beta cells. Along with successful porcine islet isolation, the need for a site that can provide optimal revascularisation leading to adequate blood glucose control is essential for long-term success [1]. We have previously shown that fetal pig pancreatic fragments (FPPFs) transplanted under the kidney capsule where these kidneys were subsequently transplanted can effectively cure both renal failure and diabetes in a single transplant [1]. Xenotransplantation of a composite kidney/FPPF graft would have the significant advantages of only requiring a single transplant and giving time for the FPPFs to mature prior to transplantation [1].

These findings are extremely relevant to both neonatal and adult islet tissues as ultimately all islet tissue is calculated on the same basis of islet equivalence and so when transplanted ends up developing to provide the same functional mass and capacity regardless of the site into which it is transplanted.

We have compared outcomes for porcine islets transplanted into three sites: kidney, liver and spleen subcapsular spaces. Porcine islets transplanted beneath the kidney capsule performed well. Transplant of islets into the kidney subcapsule showed superior results for recipient blood glucose and insulin response compared with islets transplanted into the liver and spleen. The kidney subcapsular transplant site outcomes support the findings of our companion paper investigating mouse and human islets [8].

The choice of site for the transplant has an impact on both long-term outcome and short-term complications. While the kidney has advantages shown in this study and in [8], it is unlikely to be a viable clinical option due to the small subcapsular space available in the human kidney. Groth et al were the first to transplant fetal pig islets under the kidney capsule in humans in the clinical setting [10]. However, despite having biopsiable tissue, no graft had functional porcine C-peptide release. Nevertheless, Groth et al’s study supports the potential application of composite islet/renal grafts wherein readily accessible extra islets could be co-transplanted with a kidney graft. Alternatively, the islets could be co-transplanted into the recipient kidney along with the liver, with the potential to not only provide an additional functional transplant site but also provide a more readily biopsiable indicator graft for graft surveillance. The success achieved using the liver site shows that it has potential for future clinical studies of xenotransplantation.

## Abbreviations

BGL: Blood glucose level
FPPF: Fetal pig pancreatic fragment
IEQ: Islet equivalents
